# Are aggressive epithelial cancers ‘a disease’ of Eutherian mammals?

**DOI:** 10.3332/ecancer.2018.840

**Published:** 2018-06-04

**Authors:** Miguel H Bronchud

**Affiliations:** GenesisCare Corachan Clinic, Calle Buigas 19, 08017 Barcelona, Spain

**Keywords:** cancer immune escape, cancer immune suppression, placental immune editing, cancer immunotherapies, immune vigilance concepts

## Abstract

Placental immune editing switches (PIES) have not evolved to prevent or to cause cancer but to make feto-maternal immune tolerance possible, which is at the very core of our placental mammalian (‘Eutherian’) nature. Aggressive epithelial cancers might be an unfortunate ‘side effect’ of this highly sophisticated biological nature. Microenvironmental properties in the placenta and decidua are thought to be a key to feto-maternal immune tolerance. Recently, in 2016–2018, we published the first human genomic and epigenomic evidence of similar gene expression profiles in immune regulatory genes in cancer (primary lobular infiltrating breast cancer and ipsilateral axillary metastatic lymph nodes) and both placenta and decidua of the same young patient with breast carcinoma during pregnancy. These findings led us to speculate that ectopic expression, or repression, of ‘PIES’ might be used by cancer cells during carcinogenesis or cancer progression to elude immune vigilance in spite of tumour-associated antigens or evolving neo antigenic landscapes. Cancers are well known to frequently express embryonic antigens, such as carcinoembryonic antigen, used as cancer markers and detectable in the blood circulation, or to express ectopic hormones. Why should cancer cells *invent de novo* complex new immune suppression mechanisms, if they could simply use* innate ones* developed during the long-term evolution of placental mammals in order to hide fetal paternal antigens from the mother’s own immune system?

Monotremata (Prototheria-like Echidnas or Platypus Ornithoryncus) are nonplacental egg-laying mammals and, in spite of rudimentary breast epithelial ducts and lobules, they are seldom reported to suffer from aggressive breast cancers.

## Introduction

Eutheria (or Placentalia) is the most taxonomically diverse of three branches of mammals, the other two being Metatheria (or Marsupialia) and Prototheria (or Monotremata) [1]. At present, placental mammals are a rather diverse group, with over 4000 described species, mostly rodents and bats. Humans are, of course, placental mammals (Eutheria).

Some 15 million years following dinosaur extinction, dated approximately 65 million years ago, there were already 18 extant orders of placental mammals on Earth, along with some 17 other mammalian orders that are now extinct. This very rapid evolution was theoretically facilitated by the disappearance of dinosaurs and less competition for natural habitats and food. Relatively little attention has been paid so far to the apparent fact that aggressive epithelial cancers (with aggressive local invasion and metastatic disease) are more often observed, and more frequently, the cause of natural death in Eutherians than in nonplacental mammals (such as present day Australian Echidnas or Ornythorincus Platypus), or indeed in any other vertebrates (including fish, reptiles and birds) or invertebrates [2–5]. All of them (including both invertebrates and vertebrates) develop neoplastic disease, but it seems to be placental mammals which are most likely to be affected and killed by aggressive epithelial cancers. This does not mean to say that all placental mammals suffer from aggressive epithelial carcinomas with the same frequency [6]**.** This is not the case, as the average range of cancer related natural deaths can be 5% (or even lower in some unusual creatures like underground naked mole rats) to up to 50% (or even more in some laboratory inbred rodent models).

For example, Ferris *et al* [6] recently identified accelerated DNA regions (ARs) in the elephant, hibernating bat, orca, dolphin, naked mole rat and 13-lined ground squirrel lineages in mammalian conserved regions, uncovering ∼33,000 elements that bind hundreds of different regulatory proteins in humans and mice. ARs in the elephant, the largest land mammal, are uniquely enriched near elephant DNA damage response genes.

However, it seems that placental mammals are naturally more prone to lethal epithelial cancers than most other vertebrates or invertebrates, and possibly even dinosaurs, from what is known. Radiologists X-rayed the skeleton of 10,000 dinosaur vertebrae from more than 700 museum specimens [7]. They found that a rather large part of these cancers were haemangiomas that are benign tumours of blood vessels. Other bone lesions were most likely the result of bone fractures or associated healing. Some resembled benign desmoplastic fibroma and osteoblastomas. Tumours with some degree of possibly ‘malignant features’ were only found (mainly in the tails) of Hadrosaurs and in particular the 3.5 metre species Edmontosaurus (the only one with a presumed ‘malignant tumour’ but not quite clear to be ‘metastatic’ in origin). It is obviously difficult to trace cancers in Jurassic or Cretaceous fossils because there are no soft tissue samples available, but one must also be aware of ‘pseudo pathology’: a bone fossil change may represent a post-mortem artifact rather than a true pathological lesion before death.

The frequency of cancers has been correlated with body size, with Peto reporting an unexplained ‘paradox’ [8], but not with the evolutionary aspects of placentation and the development of feto-maternal tolerance, or with the elusive concepts of immune vigilance and immune escape during carcinogenesis. The evolution of multicellularity implied the thermodynamic risk of biological disorder—loss of control on cellular differentiation or proliferation, for example—and implicitly required some natural mechanisms for the prevention of cancer. The lack of correlation between body size and cancer risk is known as ‘Peto’s Paradox’. Animals such as whales, for example, with 1,000 times more cells than humans do not usually exhibit an increased cancer risk, except perhaps beluga whales in rivers heavily contaminated by polycyclic aromatic hydrocarbons [9], suggesting that unknown natural mechanisms can suppress cancer 1,000 times more effectively than in the case for human cells. Of course, whales are not as well studied as humans, from the oncological point of view, nor are they in the same natural habitat and are not in consequence exposed to the same carcinogens (such as smoking, human carcinogenic viruses, radiation or the same types of environmental pollution and food or alcohol). But some molecular mechanisms of these ‘natural cancer suppressive processes’ have already been suggested, like increased copy numbers of tumour suppressor genes in some animal species; for example, in both African and Asian elephants, multiple copies of the tumour suppressor gene TP53 have recently been reported and this might have a cancer protective effect [10]. Other mechanisms of cancer suppression could be better methods to repair DNA mutations, or better telomeric integrity in chromosomes, or better ‘contact inhibition’ cellular mechanisms, and many others. Little has been mentioned so far on the possible role in this context of the very peculiar immune system of placental mammals, which evolved precisely to allow them to develop feto-maternal tolerance ‘in utero’.

In the past two decades, the most widely accepted theory on carcinogenesis implies clonal genetic and epigenetic changes in somatic cells that can result in at least six ‘hallmarks of cancer’ conferring certain evolutionary competitive advantages to mutating cancer cellular clones [11, 12].

Peto’s Paradox [13–15] refers to the apparent absent correlation between body size, longevity and cancer across species. Caulin and Maley [8] reviewed, in 2012, some of these issues raised by Peto’s paradox and some possible molecular explanations, including some almost anecdotal ones like, for example, cell contact inhibition that has been noted to differ between human, mouse and naked mole-rat (highly resistant to cancer). In conclusion, over the past four decades and since the original discovery of oncogenes, much has been learnt about the clonal development of cancers by the inherited (germline mutations predisposing to some familial cancers) or environmental and sporadic accumulation of activating mutations and inactivating deletions in key regulatory cellular genes (oncogenes and tumour suppressor genes, for example) which can lead specific target cells to acquire the six or seven classical ‘malignant phenotype hallmarks’. These findings have frequently translated into the clinical development of targeted drugs [16] (like small molecular weight Tyrosine Kinase Inhibitors, for example, or large monoclonal antibodies to ‘druggable targets’ on cell surface receptors). But the overall cancer pathogenesis picture is not yet complete and sequential *field models* to explain ‘sequential tissue field-related carcinogenesis’ have also been proposed (Bronchud [17], Spencer and Bronchud [18]). Key carcinogenic mutations in any cellular clone are mathematically more likely to occur in the background of more mutations in any given ‘tissue field’ than in single cells or in total cellular isolation [17, 18]. Moreover, the recent clinical development of powerful immune therapies (globally known as ‘immune check-point inhibitors’) has returned an interest in the immunological aspects of cancer control and immune escape. It is in this complex context of ‘cancer micro environmental fields’ and ‘local immune control mechanisms during carcinogenesis and cancer progression’ where our recent placental immune editing switches (PIES) hypothesis can be of potential help to better understand the cancer progression mechanisms, and how to prevent them or treat them most appropriately. This PIES hypothesis [19, 20] is not meant ‘to substitute’ the established clonal evolution theory of carcinogenesis, but ‘to complete’ it.

## Placental immune editing switches: preliminary genomic and epigenomic evidence from one single clinical and pathological clinical case

Recently, in 2016–2018, we published [19, 20] the first human genomic (in 750 immune-related genes with a Nanostring Technologies panel) and epigenomic (CpG island methylation patterns) evidence of similar immune regulatory gene expression patterns in both placenta (placenta and decidual tissue) and cancer (both primary and metastatic) [19, 20]. Similar epigenetic findings were also recently reported by Nordor *et al* [21] by using placental materials (but not uterine decidual tissues) from different human pregnancies, and comparing epigenetic CpG islands methylation patterns with ‘unrelated’ colon carcinomas (not derived from the same pregnant individuals). The same authors also suggest the existence of ‘placental immune switches’ and postulate a possible ‘time window’ (as the most likely time period for the methylome alterations) at the end of the first trimester, when cytotrophoblasts isolated from the placenta come into contact with maternal blood. However, at least theoretically, the immune escape phenomenon should first occur much earlier in the uterine decidual micro environment, in order to allow for normal blastula implantation and full embryonic development (e.g., in the first week or two of gestation), without immune rejection. Males, of course, do not have a uterus, or potentially a placenta, but most immune regulatory genes are not on the Y-chromosome, and some of these genes are also on the X-chromosome, which both males and females share.

The placenta is a unique organ evolved probably not only to nourish but also to protect the fetus from pathological environmental agents (chemical and microbiological) and from the mother’s immune system by the concerted action of many different immune cell types [22–26].

Extravillous trophoblast cells are regarded as the very first differentiated cell type of the embryo and these extraordinary cells resemble cancer cells in their capacity for proliferation, migration and establishment of blood supply [22–26], but under physiological circumstances they do not behave like a clinical cancer, and when they rarely do (usually after birth or late gestation) they are abnormal cells with several cancer-prone mutations and are called choriocarcinomas. Different mammalian species have often different types and shapes of the uterus, and their different anatomy and physiology are naturally reflected by different genomic patterns of gene expression [27]. Molecular circuits shared by placental and cancer cells and a case for positive pleiotropy between the endometrial and malignant phenotypes have been suspected for some time [28, 29].

The tens of different immune related genes similarly over or under expressed in cancer and placental microenvironments—that we recently described by applying Nanostring genomics [19]—or the hundreds of epigenetic CpG islands methylation similarities [20] between malignant tissues (primary breast cancer and metastatic lymph node) and placental/decidual tissues are unlikely to be due to a ‘single regulatory cell or pathway’, but are more likely to be the combined result of specific gene expression changes in several different immune cell types—such as macrophages, natural killer cells, dendritic antigen-presenting cells, Tregs and other T-cells or B-lymphocytes, specialised endothelial cells, myeloid-derived suppressor cells and perhaps even some fibroblasts—with regard to differential gene expression of cytokines, receptors, transcription factors, and other immune regulatory genes or regulatory RNAs. Our genomic mRNAs analysis, for example, using Nanostring PanCancer Immune Kit [19] showed that 178 genes were upregulated while 146 genes downregulated in metastatic lymph node versus normal lymph node of the same patient, and 258 immune regulatory genes were upregulated and 44 genes downregulated [19] in breast cancer tissue versus normal breast tissue in the same patient [19].

The fact that, when carefully comparing CpG island methylation of all immune regulatory genes and their known promotors [20], ‘Non-Self’ tissues in this one single clinical case of breast cancer (malignant tissues and placenta/decidua) cluster together closer than ‘Self’ tissues (normal breast and normal lymph node) is mathematically unlikely to be a chance phenomenon [20], but could represent a first example of a more general rule, although this will of course require further documentation in other clinical and pathological cases, and both suitable experimental and clinical models.

Cyclosporine A and tacrolimus are two potent immunosuppressant drugs initially developed to prevent graft rejection in patients transplanted with allogeneic kidneys or hearts and are known to inhibit the nuclear factor of activated T-cells (NFAT) pathway [30, 31]. Thus, the markedly increased CpG island methylation (and decreased gene expression) of NFATc genes 1 and 2 (Figure 4 and Table 2 in [20]), which we have found in the primary breast cancer of our patient (but not in the metastatic lymph nodes), *could result in a potent localised ‘cyclosporine-A like immunosuppressive effect’* [20].

Can cancers micro environments provide loco-regional immune suppression and cause our immune system to confuse ‘Self’ from ‘Non-Self’? Because we have shown that at least some cancers [19, 20] can reproduce in their tumour micro environments the potent immune suppressive biological effects of ‘cyclosporine-like drugs’, it is possible that direct intra tumoural injections of interleukin-2 (IL-2) might locally reverse this immune suppression and reduce tumour volume [32]. Indeed, this has been documented in a number of experimental and clinical models since the introduction in 1980s of recombinant human IL-2 and was well reviewed by Den Otter *et al* [32].

**Figure 1.**
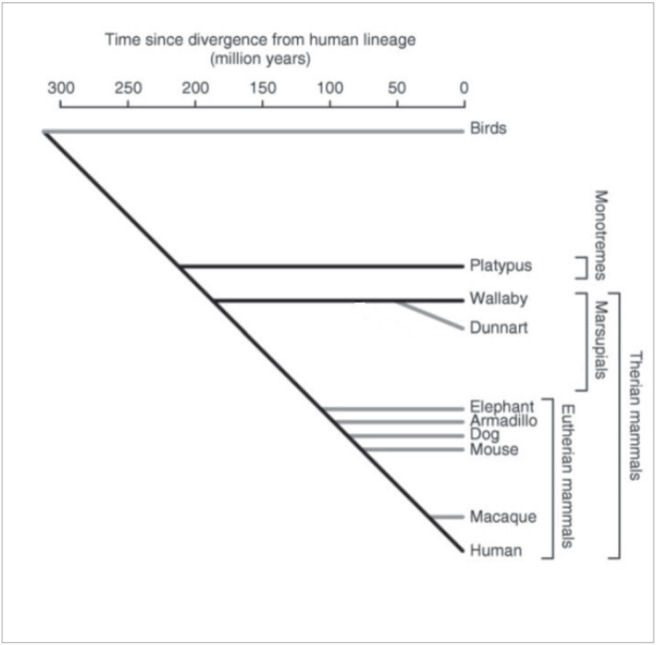
Schematic evolutionary divergence of nonplacental and placental mammals (adapted from Wakefield and Graves [34] with kind permission).

In this context, it is interesting that a novel sub-family of putatively nonclassical major histocompatibility complex class I genes that are specific to marsupials and monotremes has been recently identified and denoted as *UT* [33]. This gene family was present in the ancestral mammal and is found in marsupials and monotremes, but has been lost from the Eutherian lineage. The function of this *UT* family of genes is as yet unknown, but their predicted structure may suggest presentation of antigens to T-cells. Although not much is known on their genomics and epigenomics, marsupials and monotremes diverged from Eutherian mammals some 180 and 210 million years ago (Figure 1 and [34]), respectively, and nonfunctional sequences are expected to have diverged beyond recognition. Approximately 34% of the marsupial sequence and 14% of the platypus sequence were alignable with the human genome [34], compared with 45–75% for Eutherian mammals. A large number of short interspersed nucleotide elements have been found to be present in the platypus sequence [34].

Obviously, a lot more needs to be learnt on the molecular mechanisms responsible for ‘Self versus Non-self’ recognition in cancer biology, and also in other systems. For example, CRISPR System—a very powerful gene editing tool naturally developed to protect bacteria and archaea from invasion by phage and plasmid DNA which is now also employed in on-going sophisticated Medical gene therapy experiments—has been recently shown to distinguish ‘Self from Non-Self’ through a clever genetic interference pathway that uses the base-pairing potential of crRNAs not only to specify a ‘Non Self’ target but also to spare the ‘Self’ bacterial chromosome from interference [35].

## Conclusions

If cancer kills, it is because it deceives our body and immune system, which allows it to grow and spread. Nature would have evolved better cancer suppression mechanisms if species required them for survival and multiplication. PIES have not evolved to prevent or to cause cancer but to allow for feto-maternal immune tolerance which is at the very core of our placental mammalian nature. Aggressive epithelial cancers might be an unfortunate ‘side effect’ of this highly sophisticated biological nature, obviously not ‘predicted’ by nature because human average life expectancy was 45–50 years old until just over one century ago, and this age was in any case usually more than enough to allow procreation.

Following the demonstration, by our recently published clinical case [19, 20], of extensive genomic and epigenomic similarities between the breast cancer and metastatic axillary lymph nodes and placental/decidual immune regulatory micro environments, time has come to bring the immune component into the frontline of the discussion on the parallelisms between malignancy of cancers and some aspects of placental development with particular reference to tissue invasion, angiogenesis and growth. This ‘immune escape aspect’ has potentially very important evolutionary implications to the extent that aggressive epithelial cancers, like those most prevalent and lethal human ones (e.g. breast, colorectal, prostate, lung, gastric, ovarian, bladder, kidney, etc.), could be an unexpected and unwanted long-term consequence of our own mammalian placental (Eutherian) nature. Nature (probably some 150–200 million years ago) had ‘the choice’ to continue along the ‘oviparous path’, but the complex and several months long gestation of sophisticated animals such as humans was likely to impose ‘super eggs’ with a huge size in order to provide for adequate nourishment and protection to the fertilised egg until birth. Chicken eggs are already very large in size in comparison to the microscopic size of their only fertilised egg: large enough to protect and feed the embryo until it becomes a viable chick and hatching occurs. A human egg/human zygote, for example, has a mass of between 0.00177 and 0.0042 mg. The normal weight of a human baby who reaches full term between 37 and 40 weeks is 2.7 kg–4.1 kg (6–9 lbs), with an average weight of 3.5 kg (7.7 lbs).

This would probably have meant a huge ‘human’ egg, incompatible with our relatively narrow pelvis, for example. Instead, natural evolution opted for a different, more audacious mechanism: the fertilised egg or its blastula would engage into tissue invasion of the mother’s womb to feed directly from the mother like a parasite, but coupled to immediate immune escape from maternal immune mechanisms, to allow for nonrecognition of paternal allo-antigens. Such complex plans required several epigenetic and genomic mechanisms of placental immune editing which came at a price: the ability of neoplastic cells formed by inherited germ-line and acquired gene mutations and deletions to escape from immune vigilance and potentially lead to malignancy.

The problem, of course, is that we still do not understand these cumbersome immune regulatory pathways nor do we have yet a full knowledge of the pathways leading to ‘PIES’. Hence, there is a need to investigate them both in the ‘uterine-embryo implantation models’, in different species of placental and nonplacental mammals, and in experimental and clinical carcinogenesis or cancer progression models. Even today, one of the main reasons for the fertility treatments like *in vitro* fertilisation fail is that embryos do not implant. Evolution invented some 150 M years ago a full molecular ‘entrance exam’ set by the womb that an embryo needs to pass in order to implant. It is not unreasonable to assume that besides proteases (like trypsin) to break the natural endometrial epithelial barrier, many other genes are needed to allow effective penetration of the early embryo and efficient embedding and attachment while at the very same time hiding paternal genes from the maternal immune system. These batteries of over and under expressed genes are part of the natural gene programmes of placental mammals and we speculated can be ectopically expressed or repressed during epithelial carcinogenesis. Recently, published work in egg laying vertebrates sequencing RNA from single cells over time using complex algorithms and sophisticated genomics on several egg models (in both frogs and zebra fish) are already helping us to better understand how early embryo development has been designed by evolution to create each new cell type as a result of complex gene expression dynamics [36]. Similar approaches in mammalian placental models, if technically feasible and reproducible, might perhaps also help us to understand how the blastula implantation process (and formation of the very first differentiated epithelium, that is the embryo ‘trophoblast early epithelial layer’), can change the expression of all relevant immune regulatory genes in the uterine decidual micro environment to allow for immune escape.

## Conflicts of interest

I declare I have no conflicts of interest.

## Authors’ contributions

Miguel Hernández-Bronchud is the only author of this review.
